# Chronic Health Conditions Among US Veterans Discharged From Military Service for Misconduct

**DOI:** 10.5888/pcd15.180238

**Published:** 2018-10-11

**Authors:** Emily Brignone, J.D. Fargo, R.K. Blais, A.V. Gundlapalli

**Affiliations:** 1 Center for Health Equity Research and Promotion, VA Pittsburgh Healthcare System, Pittsburgh, Pennsylvania; 2 Informatics, Decision Enhancement, and Analytic Sciences Center, VA Salt Lake City Health Care System, Salt Lake City, Utah; 3 Department of Psychology, Utah State University, Logan, Utah; 4 Departments of Internal Medicine and Biomedical Informatics, University of Utah School of Medicine, Salt Lake City, Utah

## Abstract

Veterans who are discharged from military service due to misconduct are vulnerable to negative health-related outcomes, including homelessness, incarceration, and suicide. We used national data from the Veterans Health Administration for 218,608 veterans of conflicts in Iraq and Afghanistan that took place after the events of September 11, 2001, to compare clinical diagnoses between routinely-discharged (n = 203,174) and misconduct-discharged (n = 15,433) veterans. Misconduct-discharged veterans had significantly higher risk for all mental health conditions (adjusted odds ratio [AOR] range, 2.5–8.0) and several behaviorally linked chronic health conditions (AOR range, 1.2–5.9). Misconduct-discharged veterans have serious and complex health care needs; prevention efforts should focus on behavioral risk factors to prevent the development and exacerbation of chronic health conditions among this vulnerable population.

## Objective

Approximately 15% of recent US veterans were discharged from military service for misconduct (1). Compared with routinely-discharged veterans, misconduct-discharged veterans have high risk for incarceration, homelessness, and suicide (2–4). Studies have identified mental health disparities among this population (5), but how misconduct discharge relates to risk for chronic health conditions is unknown. Understanding the prevalence of chronic health conditions among this vulnerable population is important for understanding treatment needs and for identifying opportunities to integrate chronic disease prevention into clinical care. The purpose of this study was to compare comorbidities between misconduct-discharged and routinely-discharged US veterans.

## Methods

Data for this study were extracted from national Veterans Health Administration (VHA) databases and included records from 2006 through 2015 for 218,608 active-duty veterans of conflicts in Iraq and Afghanistan that took place after the events of September 11, 2001 (9/11). Demographic variables were age, sex, race/ethnicity, educational status, marital status, branch of service, rank, and type of discharge. Clinical data included diagnoses assigned by VHA providers during clinical encounters. Five years of retrospective follow-up data were included for each veteran, beginning on the date of each veteran’s initial VHA encounter. Analyses were conducted with R (6) and Stata (7) software through the VA’s secure VINCI workspace (8). The study was approved by the VA Salt Lake City Health Care System and the University of Utah institutional review boards.

*Discharge type*. Interservice Separation Codes, which indicate the circumstances related to separation from military service, were classified (5). Those with a “routine” or “misconduct-related” classification were included in this study.

*Health status*. The enhanced Elixhauser comorbidity index algorithm was used to create a set of 31 binary physical and mental health diagnostic outcomes, indicating the presence or absence of major chronic health conditions (9). Veterans with one primary diagnosis or more within a given category were considered to be positive. Certain outcomes categories (eg, human immunodeficiency virus (HIV), peptic ulcer disease, weight loss), were self-explanatory. For the rest, the HCUP Clinical Classification System (10) was used to create meaningful subcategories that describe the types of conditions that comprise each outcome. Subcategories were not exhaustive but provided high coverage of common conditions.

*Data analysis*. We computed descriptive statistics for all study variables. Health status variables served as binary outcomes in a series of Firth logistic regression models, which were used because of the low prevalence of certain conditions (11). The primary predictor variable was discharge type (routine vs misconduct). Covariates were age, sex, education status, marital status, race/ethnicity, branch of service, and rank. We computed separate models for men and women. Because of the large number of statistical tests conducted, an α level of .01 was used. We calculated adjusted odds ratios (AORs) and 99% confidence intervals. We computed subcategory frequencies for outcomes in which significant differences were found.

## Results

Compared with routinely-discharged veterans, misconduct-discharged veterans had lower levels of education and more were unmarried, served in the Army, and were of enlisted rank (Table 1). At the beginning of follow up, the average age of the sample was 26.7 years for misconduct-discharged veterans and 31.6 years for routinely-discharged veterans.

**Table 1:** Demographic and Military Service Characteristics of Active Duty Veterans of Post–9/11 Conflicts in Iraq and Afghanistan (N = 218,608), Veterans Health Administration, 2006–2015

Characteristic	Women^a^		Men^a^	
	Routine (N = 20,835)	Misconduct (N = 1,263)	Routine (N = 182,339)	Misconduct (N = 14,170)
**Mean age at first encounter, y (SD)**	31.5 (8.4)	26.7 (5.2)	31.6 (8.7)	26.7 (4.6)
**Race/ethnicity**				
White	9,804 (47.1)	569 (45.1)	112,874 (61.9)	8,431 (59.5)
Black	5,938 (28.5)	488 (38.6)	26,287 (14.4)	3,463 (24.4)
Hispanic	2,431 (11.7)	118 (9.3)	20,498 (11.2)	1,490 (10.5)
Other/unknown	2,662 (12.8)	88 (7.0)	22,680 (12.4)	786 (5.5)
**Education level**				
High school/equivalent	15,912 (77.7)	1,127 (90.7)	151,753 (84.6)	13,077 (93.9)
Beyond high school	4,577 (22.3)	116 (9.3)	27,672 (15.4)	846 (6.1)
**Marital status**				
Never married	11,709 (56.4)	856 (68)	100,536 (55.2)	9,685 (68.4)
Married	7,631 (36.7)	356 (28.3)	77,184 (42.4)	4,292 (30.3)
Divorced/other	1,430 (6.9)	46 (3.7)	4,402 (2.4)	177 (1.3)
**Branch of service**				
Army	8,041 (38.6)	720 (57.0)	74,437 (40.8)	8,674 (61.2)
Marines	1,832 (8.8)	44 (3.5)	46,560 (25.5)	1,375 (9.7)
Air Force	5,389 (25.9)	234 (18.5)	26,384 (14.5)	1,345 (9.5)
Navy/Coast Guard	5,573 (26.7)	265 (21.0)	34,956 (19.2)	2,776 (19.6)
**Rank**				
Enlisted	18,994 (91.2)	1,212 (96.0)	169,982 (93.2)	13,874 (97.9)
Officer/Warrant	1,841 (8.8)	51 (4.0)	12,357 (6.8)	296 (2.1)

Abbreviations: 9/11, the events of September 11, 2001; SD, standard deviation.

a Values are no. (%) unless otherwise indicated.

Misconduct discharge was significantly associated with higher risk for several conditions (Table 2). Among men, misconduct discharge was associated with significantly higher odds for cardiac arrhythmias (AOR = 1.2), paralysis (AOR = 2.1), neurological disorders (AOR = 2.1), chronic pulmonary disease (AOR = 1.3), liver disease (AOR = 1.6), peptic ulcer disease (AOR = 1.5), acquired immunodeficiency syndrome (AIDS)/HIV (AOR = 3.3), weight loss (AOR = 1.4), and fluid and electrolyte disorders (AOR = 1.7).

**Table 2:** Frequencies and Adjusted Risk for Clinical Comorbidities Over 5 Years of Follow-Up Among Active Duty Veterans of Post–9/11 Conflicts in Iraq and Afghanistan (N = 218,608), Veterans Health Administration, 2006–2015

Elixhauser Category	Women			Men		
	Routine (N = 20,835)	Misconduct (N = 1,263)	AOR^a^ (99% CI)	Routine (N = 182,339)	Misconduct (N = 14,170)	AOR^a^ (99% CI)
	No. (%)			No. (%)		
Congestive heart failure	34 (0.2)	3 (0.2)	2.13 (0.48–9.54)	324 (0.2)	28 (0.2)	1.44 (0.84–2.47)
Cardiac arrhythmias	464 (2.2)	30 (2.4)	1.03 (0.61–1.72)	4,002 (2.2)	381 (2.7)	1.21 (1.05–1.41)
Valvular disease	76 (0.4)	4 (0.3)	1.20 (0.33–4.30)	460 (0.3)	29 (0.2)	0.97 (0.58–1.62)
Pulmonary circulation disorders	37 (0.2)	4 (0.3)	2.41 (0.62–9.36)	220 (0.1)	23 (0.2)	1.32 (0.72–2.43)
Peripheral vascular disorders	40 (0.2)	2 (0.2)	1.64 (0.29–9.14)	354 (0.2)	28 (0.2)	1.58 (0.93–2.70)
Hypertension, uncomplicated	1,658 (8.0)	86 (6.8)	1.32 (0.97–1.81)	21,623 (11.9)	1,299 (9.2)	1.02 (0.94–1.10)
Hypertension, complicated	15 (0.1)	1 (0.1)	1.97 (0.18–21.00)	200 (0.1)	15 (0.1)	1.30 (0.64–2.67)
Paralysis	24 (0.1)	1 (0.1)	0.95 (0.10–8.90)	269 (0.1)	42 (0.3)	2.05 (1.30–3.24)
Other neurological disorders	212 (1.0)	43 (3.4)	2.84 (1.77–4.57)	1,972 (1.1)	376 (2.7)	2.14 (1.82–2.50)
Chronic pulmonary disease	1,642 (7.9)	143 (11.3)	1.35 (1.05–1.73)	8,993 (4.9)	992 (7)	1.31 (1.20–1.44)
Diabetes, uncomplicated	389 (1.9)	17 (1.3)	1.03 (0.53–2.23)	4,285 (2.4)	196 (1.4)	1.05 (0.86–1.28)
Diabetes, complicated	30 (0.1)	0 (0.0)	0.41 (0.01–17.00)	457 (0.3)	28 (0.2)	1.45 (0.86–2.44)
Hypothyroidism	826 (4.0)	42 (3.3)	1.07 (0.70–1.64)	1,783 (1.0)	104 (0.7)	0.88 (0.67–1.16)
Renal failure	27 (0.1)	1 (0.1)	1.63 (0.17–15.76)	556 (0.3)	43 (0.3)	1.16 (0.74–1.80)
Liver disease	103 (0.5)	12 (10.0)	2.52 (1.10–5.81)	1,519 (0.8)	168 (1.2)	1.58 (1.27–1.98)
Peptic ulcer disease	42 (0.2)	8 (0.6)	4.22 (1.51–11.83)	411 (0.2)	56 (0.4)	1.54 (1.04–2.29)
AIDS/HIV	11 (0.1)	2 (0.2)	3.45 (0.48–25.02)	237 (0.1)	88 (0.6)	3.30 (2.33–4.65)
Lymphoma	19 (0.1)	0 (0.0)	0.79 (0.19–33.61)	171 (0.1)	13 (0.1)	1.17 (0.55–2.50)
Metastatic cancer	15 (0.1)	3 (0.2)	5.87 (1.03–33.84)	114 (0.1)	13 (0.1)	1.56 (0.70–3.47)
Solid tumor without metastasis	231 (1.1)	18 (1.4)	1.96 (1.01–3.84)	1,119 (0.6)	70 (0.5)	1.10 (0.79–1.54)
RA/collagen vascular diseases	292 (1.4)	14 (1.1)	0.98 (0.48–2.00)	1,030 (0.6)	90 (0.6)	1.16 (0.86–1.56)
Coagulopathy	44 (0.2)	5 (0.4)	2.20 (0.85–7.47)	338 (0.2)	26 (0.2)	0.98 (0.57–1.67)
Obesity	1,854 (8.9)	125 (9.9)	1.11 (0.86–1.44)	10,164 (5.6)	686 (4.8)	0.81 (0.73–0.91)
Weight loss	102 (0.5)	15 (1.2)	2.13 (1.02–4.43)	567 (0.3)	82 (0.6)	1.41 (1.02–1.95)
Fluid and electrolyte disorders	170 (0.8)	15 (1.2)	1.57 (0.77–3.20)	862 (0.5)	130 (0.9)	1.74 (1.34–2.25)
Blood loss anemia	31 (0.1)	2 (0.2)	1.35 (0.23–7.89)	26 (0.0)	2 (0.0)	1.15 (0.19–6.90)
Deficiency anemia	383 (1.8)	27 (2.1)	1.21 (0.71–2.05)	403 (0.2)	26 (0.2)	0.86 (0.50–1.48)
Alcohol abuse	604 (2.9)	221 (17.5)	5.58 (4.42–7.07)	13,557 (7.4)	3,558 (25.1)	3.44 (3.25–3.65)
Drug abuse	363 (1.7)	215 (17)	8.04 (6.22–10.40)	7,666 (4.2)	3,339 (23.6)	5.14 (4.82–5.49)
Psychoses	308 (1.5)	63 (5.0)	2.84 (2.40–3.34)	2850 (1.6)	838 (5.9)	2.46 (2.34–2.60)
Depression	8,011 (38.4)	855 (67.7)	2.97 (2.02–4.38)	73,047 (40.1)	9,566 (67.5)	2.95 (2.64–3.30)

Abbreviations: 9/11, the events of September 11, 2001; AIDS, acquired immunodeficiency syndrome; AOR, adjusted odds ratio; CI, confidence interval; HIV, human immunodeficiency virus; RA, rheumatoid arthritis.

a Odds adjusted for age, race/ethnicity, marital status, education, branch of service, and rank.

Among women, misconduct discharge was associated with significantly higher odds for neurological disorders (AOR = 2.8), chronic pulmonary disease (AOR = 1.4), liver disease (AOR = 2.5), peptic ulcer disease (AOR = 4.2), metastatic cancer (AOR = 5.9), solid tumor without metastasis (AOR = 2.0), and weight loss (AOR = 2.1).

Men and women with a misconduct discharge had higher odds for alcohol abuse, drug abuse, depression, and psychosis (AOR range, 2.5–8.0). For mental health–related outcomes, the presence of diagnoses in multiple subcategories was more common among misconduct-discharged veterans, indicating higher comorbidity (Table 3).

**Table 3:** Frequencies and Descriptions for Subcategories Composing Selected Categories of Comorbidity Over 5 Years of VHA Follow-Up Among Active Duty Veterans of Post–9/11 Conflicts in Iraq and Afghanistan (N = 218,608), Veterans Health Administration, 2006–2015

Elixhauser Category	Women, %^a^		Men, %^a^	
	Routine	Misconduct	Routine	Misconduct
**Cardiac arrhythmias**				
Cardiac dysrhythmias	—		96.5	94.8
Conduction disorders	—		5.8	8.4
**Paralysis**				
Other paralysis	—		85.5	88.1
Hemiplegia	—		14.1	7.1
Other central nervous system disorder	—		5.9	11.9
**Other neurological disorders**				
Convulsions	63.2	79.1	71.1	80.6
Epilepsy	31.1	51.2	31.9	31.4
Hereditary and degenerative nervous system conditions	25.5	4.7	13.1	5.1
**Chronic pulmonary disease**				
Other and unspecific asthma	67.7	53.1	58.7	54.3
Other chronic pulmonary disease	30.9	49.0	32.8	43.5
Chronic airway obstruction; not otherwise specified	8.5	6.3	13.2	9.5
**Liver disease**				
Hepatitis	37.9	66.7	35.9	61.9
Other liver diseases	58.3	25.0	58.7	31.5
Alcohol — related disorders	5.8	8.3	12.7	16.1
**Metastatic cancer**				
Malignancy without site specification	53.3	33.3	—	
Secondary malignancy of lymph nodes	26.7	66.7	—	
Secondary malignancy of bone	13.3	0	—	
Secondary malignancy of brain/spine	6.7	33.3	—	
Secondary malignancy of liver	6.7	0	—	
**Solid tumor without metastasis**				
Cancer of breast	32.5	22.2	—	
Cancer; other primary	29.0	38.9	—	
Cancer of uterus and cervix	13.9	16.7	—	
Cancer of skin	11.3	5.6	—	
Cancer of ovary and other female genital organs	9.1	5.6	—	
Cancer of bronchus; lung	2.2	5.6	—	
**Fluid and electrolyte disorders**				
Hypovolemia	—		47.0	59.2
Hypopotassemia	—		30.7	26.9
Hyperpotassemia	—		13.7	6.9
**Drug abuse**				
Drug use disorder, unspecified	71.1	68.8	70.3	71.2
Opioid use disorder	15.7	21.9	24.0	25.4
Cannabis use disorder	30.0	40.9	28.0	37.5
Cocaine use disorder	11.8	26.5	11.5	19.4
Amphetamine use disorder	7.4	8.8	6.0	9.3
**Psychosis**				
Schizophrenia and other psychotic disorders	48.4	66.7	58.0	74.6
Depressive disorders (with psychotic features)	47.7	28.6	36.6	21.6
Bipolar disorders (with psychotic features)	6.8	12.7	6.1	9.7
**Depression**				
Post-traumatic stress disorder	52.1	63.7	73.3	76.0
Depressive disorders (without psychotic features)	69.4	72.0	45.6	55.6
Adjustment disorders	30.1	31.2	29.3	29.3

Abbreviation: 9/11, the events of September 11, 2001.

a Percentage of veterans within each Elixhauser comorbidity category who have a diagnosis in the given subcategory. Subcategory tabulations are presented for selected categories where significant differences between discharge types were observed. The most common subcategories were selected for presentation. Totals may exceed 100% due to co-occurring conditions within the same Elixhauser category.

## Discussion

Misconduct-discharged veterans experience significant disparities in several chronic health conditions, many of which are behaviorally linked, including chronic pulmonary disease, liver disease, and AIDS/HIV. These disparate risks likely relate to the unique mental and behavioral health characteristics of this population. Specifically, misconduct may occur as a result of poor executive functioning and decision making while in service, sometimes caused by unmet or unrecognized mental health needs. Following discharge, these factors may continue to drive health and social outcomes.

Targeted prevention efforts in the VHA may help mitigate the vulnerabilities of this population. Integrating chronic disease prevention into the mental health and social services that misconduct-discharged veterans are likely to use may be an effective strategy. In addition, transitioning the care of misconduct-discharged veterans from acute settings into integrated primary care settings such as patient-centered medical homes may enable the VHA to more efficiently meet their complex health needs.

This study has limitations. Diagnostic codes may contain errors, and codes may be applied inconsistently across providers. Certain findings, such as high incidence of rare conditions such as cardiac arrhythmias, merit further study. This sample is based on users of VHA care, and there may be differences between those who do and do not access VHA services. We are also unable to assess non-VHA health service use, and we did not include information relating to service-connected disability benefit level, which may factor into the decision to use VHA services. Future research should assess symptoms among a broader sample of veterans that include older cohorts, because disparities may multiply with increasing age.

Results from this study demonstrate that misconduct discharge can be a marker for behaviorally linked chronic health conditions, and they underscore the reciprocal relationship between mental and physical health in both military and civilian settings. Strategies for improving outcomes among this vulnerable population may include targeted screening for risky health behaviors and early signs of chronic health conditions and the integration of preventive care with mental and behavioral care.
